# Genetic and Antigenetic Characterization of the Novel Kotalahti Bat Lyssavirus (KBLV)

**DOI:** 10.3390/v13010069

**Published:** 2021-01-06

**Authors:** Sten Calvelage, Niina Tammiranta, Tiina Nokireki, Tuija Gadd, Elisa Eggerbauer, Luca M. Zaeck, Madlin Potratz, Claudia Wylezich, Dirk Höper, Thomas Müller, Stefan Finke, Conrad M. Freuling

**Affiliations:** 1 Institute of Diagnostic Virology, Friedrich-Loeffler-Institut (FLI), 17493 Greifswald-Insel Riems, Germany; sten.calvelage@fli.de (S.C.); claudia.wylezich@fli.de (C.W.); dirk.hoeper@fli.de (D.H.); 2 Finnish Food Authority, Research Department, Virology Unit, Mustialankatu 3, FI-00790 Helsinki, Finland; niina.tammiranta@ruokavirasto.fi (N.T.); tiina.nokireki@ruokavirasto.fi (T.N.); tuija.gadd@ruokavirasto.fi (T.G.); 3 Institute of Molecular Virology and Cell Biology, Friedrich-Loeffler-Institut (FLI), WHO Collaborating Centre for Rabies Surveillance and Research, OIE Reference Laboratory for Rabies, 17493 Greifswald-Insel Riems, Germany; Elisa.Eggerbauer@gmx.de (E.E.); luca.zaeck@fli.de (L.M.Z.); madlin.potratz@fli.de (M.P.); Thomas.Mueller@fli.de (T.M.); stefan.finke@fli.de (S.F.); 4 Central Duties, Friedrich-Loeffler-Institut (FLI), 17493 Greifswald-Insel Riems, Germany

**Keywords:** KBLV, rabies, *Myotis brandtii*, lyssavirus

## Abstract

There is a growing diversity of bat-associated lyssaviruses in the Old World. In August 2017, a dead Brandt’s bat (*Myotis brandtii*) tested positive for rabies and based on partial sequence analysis, the novel Kotalahti bat lyssavirus (KBLV) was identified. Because the bat was in an autolyzed state, isolation of KBLV was neither successful after three consecutive cell passages on cells nor in mice. Next generation sequencing (NGS) was applied using Ion Torrent ™ S5 technology coupled with target enrichment via hybridization-based capture (myBaits^®^) was used to sequence 99% of the genome, comprising of 11,878 nucleotides (nt). KBLV is most closely related to EBLV-2 (78.7% identity), followed by KHUV (79.0%) and BBLV (77.6%), supporting the assignment as phylogroup I lyssavirus. Interestingly, all of these lyssaviruses were also isolated from bat species of the genus Myotis, thus supporting that *M. brandtii* is likely the reservoir host. All information on antigenic and genetic divergence fulfil the species demarcation criteria by ICTV, so that we recommend KBLV as a novel species within the Lyssavirus genus. Next to sequence analyses, assignment to phylogroup I was functionally corroborated by cross-neutralization of G-deleted RABV, pseudotyped with KBLV-G by sera from RABV vaccinated humans. This suggests that conventional RABV vaccines also confer protection against the novel KBLV.

## 1. Introduction

Bats (*Chiroptera*) with the unique trait of true flight are a fascinating order of mammals which encompasses more than 1200 species [1]. Beside this biological curiosity, bats are assumed or verified reservoir host for viruses including some which cause disease in humans, such as viruses from the genera Ebolavirus, Marburgvirus, Henipavirus and Lyssavirus [2,3,4,5]. However, this seemingly exaggerated importance of bats for zoonotic diseases needs careful interpretation [6]. 

Rabies was the first zoonotic disease associated with bats, and progress in research verified that the causative agents are diverse members of the lyssaviruses [7]. This genus within the family *Rhabdoviridae*, order *Mononegavirales* constitutes of a growing number of viral pathogens that have been characterized and officially classified. Currently, the lyssavirus genus includes 17 recognized and one related, unclassified virus species [8,9], which based upon genetic, immunologic, and pathologic characteristics of certain members can be assigned to at least three distinct phylogroups [10,11,12]. All lyssaviruses are assumed to be capable of causing rabies, i.e., an infection of the central nervous system that inevitably lead to lethal encephalitis [13].

In Europe, bat rabies is the only known bat transmitted zoonosis and has caused human casualties [14]. In 1977, the first human rabies case associated with a bat bite in Europe was reported in the Ukraine. Another confirmed human rabies case in Russia transmitted from a bat occurred in 1985 [15]. Bat handlers also died of EBLV-2 induced, laboratory confirmed rabies in Finland in 1985 [16] and in Scotland in 2002 [17]. In all cases, the individual infected had a history of close contact with bats and none had received vaccination against rabies. Spillover infections into terrestrial mammals with EBLV-1 were sporadically documented [18,19,20].

Initially, rabies in bats in Europe was detected in 1954. Subsequent molecular characterization demonstrated that viruses isolated from rabid European bats and associated human rabies cases were distinct from classical rabies virus (RABV) and belong to two genetically separate lyssaviruses, European bat lyssavirus type 1 (EBLV-1) and European bat lyssavirus type 2 (EBLV-2) [21]. Since 1977, more than one thousand lyssavirus infections in bats have been identified across Europe with the majority of cases in caused by EBLV-1 [22], which was mostly found associated with *Eptesicus serotinus* and *Eptesicus isabellinus*, the latter confined to Spain [23]. Bat rabies cases caused by EBLV-2 were sporadically detected in Daubenton’s (*Myotis daubentonii*) and Pond bats (*M. dasycneme*) [24]. In recent decades, the diversity of known bat lyssaviruses in Europe has expanded. West Caucasian Bat Virus (WCBV) was isolated from a single common bent-winged bat (*Miniopterus schreibersii)* in 2003 [25]. Eight years later, in 2011, yet another, highly divergent lyssavirus, termed Lleida Bat Lyssavirus (LLEBV) was discovered in a bat of the same species in Catalonia, Spain [11,26]. LLEBV was again isolated in southern France [27]. Since 2010, the new Bokeloh bat lyssavirus (BBLV) was detected in Natterer’s bats (*M. nattereri*), first in Germany [28], but later also in France and Poland [29,30,31].

In August 2017, a dead Brandt’s bat (*M. brandtii*) was found in Eastern Finland in the village of Kotalahti (62°29′30″ N, 027°47′15″ E). The bat tested positive for rabies in the fluorescent antibody test (FAT) and while virus isolation was unsuccessful, RT-PCR and subsequent phylogenetic analysis revealed that the virus differed from other known lyssaviruses and was designated as Kotalahti bat lyssavirus (KBLV) [32]. Here, we describe further attempts to genetically and functionally characterize this novel bat lyssavirus for which only sequence information was available. 

## 2. Materials and Methods

### 2.1. Rabies Tissue Culture Isolation Test (RTCIT)

Prior to virus isolation attempts at the Friedrich-Loeffler-Institute, scarce remaining brain material and material from the spinal cord of the above-mentioned bat was homogenized in MEM using the TissueLyzer (Quiagen, Hilden, Germany) and then clarified by centrifugation (1000× *g* for 3 min). Virus isolation attempts followed previously published protocols [33]. Briefly, murine neuroblastoma cells (MNA 42/13, CCLV-RIE 0229) were split and mixed with the clarified homogenate. After incubation at 37 °C and 5% CO_2_ for 72 h, cells of control dishes were fixed and stained with a rabies anti-nucleocapsid FITC-conjugated antibody (SIFIN, Berlin, Germany). Attempts to isolate virus were stopped after three consecutive passages with negative results. 

### 2.2. In Vivo Studies

For in-vivo virus isolation attempts, additionally, six 3-week-old female (BALB/C, Charles River, Sulzfeld, Germany) and six suckling mice (BALB/C, FLI in-house breeding) were inoculated with homogenized, clarified sample (5–10 µL) intra-cerebrally. Mice had access to food and water ad libitum and were monitored daily for 21 days using an established lyssavirus clinical scoring system to evaluate any behavioural changes consistent with lyssavirus infection. Brains of euthanized mice were tested by direct fluorescent antibody test (FAT) [34]. 

### 2.3. Next Generation Sequencing (NGS) and Phylogenetic Analysis

Because of limited original brain material, the precipitate from the inoculum for isolation was further used for molecular detection. The precipitate was disintegrated using the cryoPREP CP02 (Covaris, Brighton, United Kingdom) and the pulverized material was subsequently lysed in 1 mL pre-heated (56 °C) AL Buffer (Qiagen, Hilden, Germany). For RNA extraction, a threefold volume of Trizol LS (Thermo Fisher Scientific, Waltham, MA, USA) was added and the aqueous phase of the resulting mixture further processed utilizing the RNeasy Mini Kit (Qiagen, Hilden, Germany) including an on-column DNase I (Qiagen, Hilden, Germany) digestion step. In preparation for the metagenomic analysis, the sample was further treated as previously described [35]. Briefly, extracted RNA was converted into double stranded cDNA using the cDNA synthesis system kit (Roche, Mannheim, Germany) together with random hexamer primers (Roche). Afterwards, the obtained cDNA was fragmented using the Covaris M220 instrument (Covaris) and subsequently processed with the GeneRead L Core Kit (Qiagen, Hilden, Germany) and the respective IonXpress Barcode adaptors (Thermo Fisher Scientific, Waltham, MA, USA) for the generation of Ion Torrent compatible libraries. After size-selection, the resulting library L02374 was quality controlled (Agilent 2100 Bioanalyzer, High Sensitivity DNA Kit, Agilent Technologies, Santa Clara, CA, USA) and quantified (KAPA Library Quantification Kit-Ion Torrent PGM Uni; KAPA Biosystem/Roche, Basel, Switzerland). Subsequently, the library was sequenced on an Ion Torrent S5 XL instrument (Thermo Fisher Scientific) utilizing a Ion 530 chip and reagents for 400 bp sequencing (Thermo Fisher Scientific) according to the manufacturer’s instructions. 

Target enrichment was performed via hybridization-based capture using RNA baits (myBaits, Arbor Biosciences, Ann Arbor, Michigan, USA) according to the myBaits manual v.4.01 (April 2018). Briefly, a pre-designed diagnostic bait set of 80mer biotinylated oligonucleotides targeting sequences of epizootic and zoonotic viral pathogens containing about 11,500 baits specific for RABV [36] was added to the cDNA library for hybridization at a temperature of 62 °C for 16 h. The resulting biotinylated target-bait complex was bound to streptavidin-coated beads and unbound baits were washed away. An aliquot (16 µL) of the enriched lyssavirus nucleotides (nt) was subsequently amplified using the GeneRead DNA Library L amplification Kit (Qiagen, Hilden, germany) according to manufacturer‘s instructions performing 14 cycles and purified using solid-phase paramagnetic bead technology (Agencourt AMPure XP magnetic beads; Beckman Coulter, Fullerton, USA). The enriched library was again quantified before sequencing it as described above.

In order to acquire the KBLV full genome sequence, an initial de-novo assembly of raw sequencing data was conducted (454 Sequencing System Software v3.0; Roche) and the generated contigs were taxonomically assigned using BLAST^®^ and the associated NCBI nucleotide database (blastn) [37]. The so identified viral contigs were further used in a reference-based mapping (454 Sequencing System Software v3.0; Roche). The resulting KBLV genome sequence was annotated using Geneious Prime (2019.2.3; build 2019-09-24) and submitted to the European Nucleotide Archive (ENA) under the accession number PRJEB41152.

In order to phylogenetically assign KBLV within the genus lyssavirus, a multiple sequence alignment encompassing reference sequences (where available) of all 17 classified lyssavirus species (Table S1) together with the KBLV genome was conducted utilizing the MAFFT plugin (v7.388, [38]) with default settings as implemented in Geneious Prime. Subsequently, a maximum-likelihood phylogenetic tree was generated using IQ-Tree (v.1.6.5) with 100.000 ultrafast-bootstraps and enabled ModelFinder feature [39].

### 2.4. Plasmids and cDNA Cloning

The expression plasmid pCAGGS L16G comprised the sequence of the RABV vaccine strain SAD B19 [40]. pCAGGS-KBLVG was constructed by PCR amplification of the 1.6 kb KBLV G open reading frame from synthetic cDNA with the the primers 5′-TGCCCGGGTACCATGCATCGATGAGCTCGCTAGGCCTGAGACTGATCTCC-3′ and 5′-TTGTTGTGCTGTCTCATCATTTTGGCAAAGGGAAAGATGCCATTTCAAGC-3′ and insertion in the EcoRI linerized 4.7 kb pCAGGS vector [41] by Hot Fusion reaction [42].

### 2.5. G-Deleted Virus and Generation of Pseudotyped RABV

For *in trans* complementation of G gene deleted SAD ∆G GFP, in which the G ORF was replaced by the green fluorescence protein (GFP) reporter ORF [43], 3 × 10^6^ BSR T7/5 cells in a T25 cell culture flask were infected at multiplicity of infection (MOI) of 1. After 2 h of incubation at 37 °C, cells were trypsinized and the cell suspension was transferred to 6-well plates. Following over-night incubation, the infection was controlled by GFP autofluorescence and expression plasmids for G proteins of SAD B19 and KBLV (see Section 2.4) were transfected into the infected cells using the polyethylenimine technique (see below). At two days post transfection the cell culture supernatants were collected and used for neutralization assays.

*DNA transfection.* Plasmid DNA transfections into BSR T7/5 cells was performed with polyethylenimine (PEI; Sigma-Aldrich, Hamburg, Germany). Briefly, 2.5 to 5.0 × 10^5^ cells were seeded in 3.5 cm dishes. After overnight incubation, the cell culture medium was replaced by 1 mL serum free DMEM medium and 1 h later a mixture of 6µg plasmid and 9µg PEI in 800 mL DMEM was added. For preparation of the transfection solution, plasmid DNA and PEI were separately diluted in 400 µL DMEM and then mixed after 5 min incubation at room temperature (20–22 °C). After further 20 min incubation, the mix was added to the cell culture. After further 3.5 h of incubation at 37 °C, medium and transfection mix was removed and replaced by 2 mL fresh culture medium.

*Antibodies, sera and immunofluorescence microscopy.* Mouse monoclonal antibody E559 against RABV G protein have been described previously [44]. The polyclonal rabbit serum against EBLV-2 G protein was generated by immunization with an *E. coli* expressed and purified partial G protein purified MBP-EBLV-2G protein (EBLV-2G fused to Maltose binding protein (MBP)). For indirect immunofluorescence detection, on glass covers slips growing cells were fixed with 4% paraformaldehyde (PFA) in phosphate buffered saline (PBS). After 30 min incubation at room temperature, the PFA was removed and the specimens were blocked by incubation with 0.025% milk powder in PBS for 20 min at RT. Immunostainings were performed by two hours incubation with primary antibody, two wash steps with PBS and subsequent 45 min incubation with Alexa-fluorophore conjugated secondary antibodies. Images were acquired with a Leica SP5 confocal laser scan microscope (63x objective; numerical aperture: 1.4) with sequential acquisition of the fluorophores in double fluorescent specimens. Images were processed with the ImageJ software version 1.48b [45,46] 

### 2.6. Cross-Neutralization of KBLV by Human Sera

For cross-neutralization, serum samples (*N* = 27) from humans vaccinated against RABV were used. The virus neutralizing titre was initially established using the Rapid Fluorescent Foci Inhibition Test (RFFIT) with CVS-11 as test virus [47]. Briefly, sera were tested in duplicate in two-fold serial dilutions on mouse neuroblastoma cells (MNA 42/13) with a starting dilution of 1:10. The VNA titer was expressed as the reciprocal of the serum dilution showing a 50% reduction in fluorescent foci of the test virus in vitro and the exact titer was calculated using inverse interpolation. 

In this study, G-deleted RABV, pseudotyped with KBLV-G was additionally used as test virus and the RFFIT procedures followed previously published protocols [47]. Briefly, tissue culture supernatant of transfected cells (see above) was used and mixed with serum. For conversion into international units, a heterologous WHO international standard immunoglobulin (2nd human rabies immunoglobulin preparation, National Institute for Standards and Control, Potters Bar, UK) was adjusted to 0.5 IU/mL for CVS-11, and 1.5 IU/mL for KBLV. All data were log10 transformed. Graphical visualization and a simple linear regression analysis (best-fit line was performed using GraphPad Prism version 8.0.0 for Windows (GraphPad Software, San Diego, CA, USA).

## 3. Results

### 3.1. Virus Isolation from Tissue Samples Failed

Brain material from the fluorescent antibody test (FAT) and the PCR positive bat was used for virus isolation attempts. Tissue cultures after three consecutive passages were negative in the FAT. In addition, all mice survived until the termination of the observation period at day 21 post infection and brain impression smears tested negative in FAT.

### 3.2. NGS, Sequence Analysis and Phylogeny

**Figure 1:**
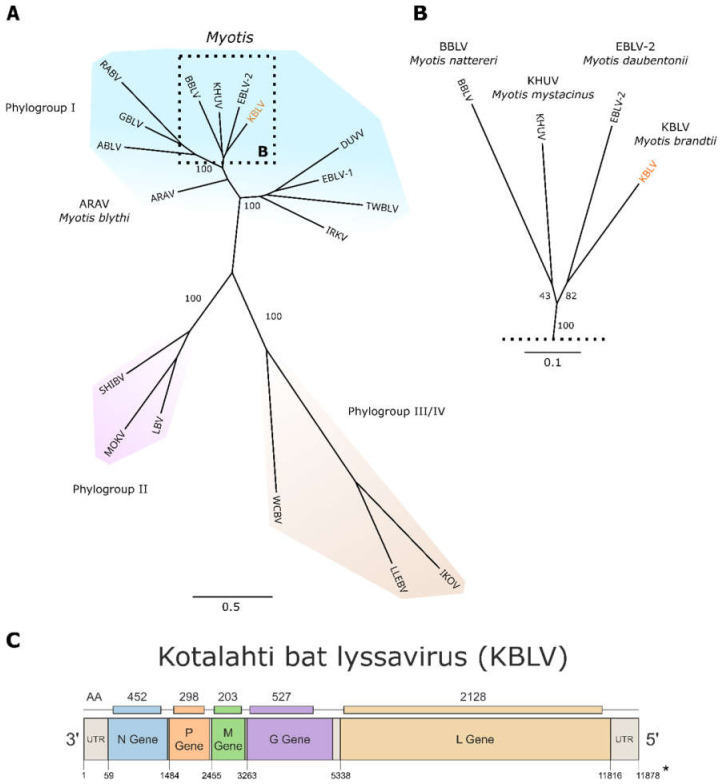
(**A**) ML-Phylogenetic tree based on full-genome sequences of representatives of the 17 different lyssavirus species and their respective affiliation to one of the four designated phylogroups (I-IV) (**B**) Enlarged part of the tree showing the lyssaviruses most closely related to KBLV and the bat species they were isolated from; (**C**) The 11,878 nt long sequence of the Kotalahti bat lyssavirus (KBLV) genome reflects the characteristic gene organization known for lyssaviruses encoding for the five viral proteins: nucleoprotein (N), phosphoprotein (P), matrix protein (M), glycoprotein (G) and the RNA-dependent RNA polymerase (L). * Sequence depth of the 5′UTR was not sufficient at the final part to verify nucleotides.

Viral RNA could be isolated from the remaining material, albeit the quality of RNA was low as shown by a high level of fragmentation (see Figure S1). Nonetheless, sequencing of the resulting cDNA library yielded 192,968 lyssavirus reads (3.4%). Because a de-novo assembly could not generate a full-genome sequence, a targeted enrichment protocol using customised biotinylated RNA-baits was used to increase the number of on-target reads, resulting in 459,263 lyssavirus reads (90.40%) covering 99.9% of the KBLV genome. The obtained genome sequence consists of 11,878 nt encoding for the five genes N, P, M, G and L, in the highly conserved order typical for lyssaviruses (Figure 1, Table S2). Only for the UTR and LTR, raw sequence information was insufficient to derive the entire sequence.

Phylogenetically, KBLV is closest related to EBLV-2 and clusters together with KHUV and BBLV (Figure 1). The genetic pairwise identity with the reference sequence of EBLV-2 (NC_009528) is 78.7%, whereas it is 79.0% and 77.6% to KHUV (NC_025385) and BBLV (NC_025251), respectively. Beside the acquisition of the KBLV genome sequence, the obtained sequencing data was also screened for host cytochrome B sequences, thereby confirming the host species as *Myotis brandtii*.

### 3.3. Expression of KBLV Proteins in Transfected Cells

**Figure 2:**
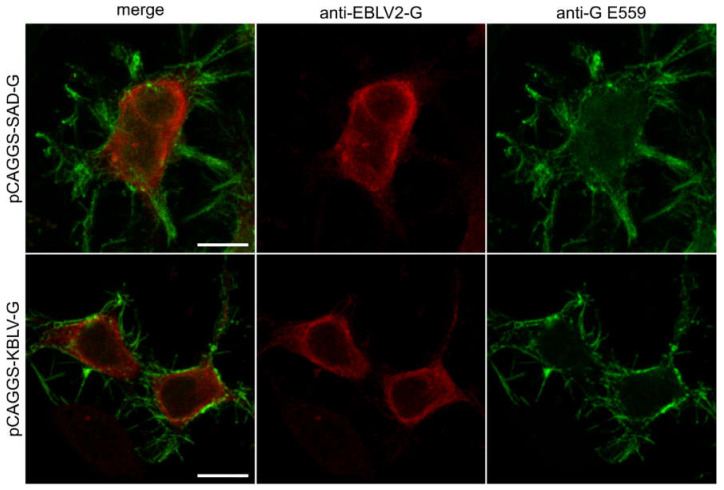
Distribution of KBLV G and RABV G protein in plasmid transfected cells: intracellular G-protein (red, stained with a rabbit-anti—EBLV-2 G polyclonal serum) and extracellular G-protein (green, stained with E559). Notably, E559 is a rabies virus-neutralizing monoclonal antibody [44] recognizing the conformational epitope of fully matured G-protein. The scale bar is 10 µm in length.

To assess expression, intracellular distribution, and antigenicity of the predicted KBLV glycoprotein, expression plasmid vectors were constructed by insertion of the respective ORF as a synthetic cDNA. At one day after plasmid transfection, the KBLV and RABV G proteins were comparably detected at the cell surface by the monoclonal antibody E559 and intracellularly by a serum raised against EBLV-2 G (Figure 2). 

### 3.4. Cross-Neutralization

**Figure 3:**
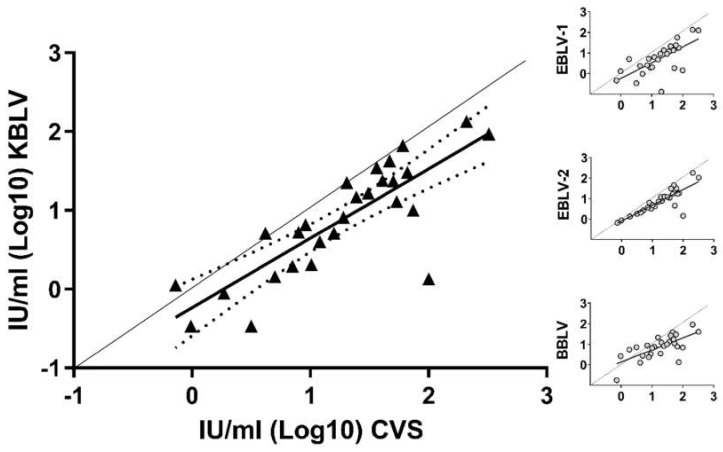
Graph showing individual concentrations of virus neutralizing antibodies as measured using CVS (*x*-axis) and RABV pseudotyped with KBLV-G as test virus (*y*-axis), plotted in double-logarithmic graphs. The 95% confidence intervals (CI; Bonferroni corrected) of the best-fit regression line are indicated (dashed lines). Insets: Comparative analyses using the same sera and EBLV-1, EBLV-2 and BBLV as test viruses ([47], Table S3).

In order to test whether sera from human RABV vaccinees would cross-neutralize KBLV, G-deleted RABV pseudotyped with KBLV-G were used. All sera from human vaccinees cross-neutralized the supernatant of RABV pseudotyped with KBLV-G. The neutralizing activity of sera was lower against KBLV than against the standard RABV test virus (Challenge Virus Standard, CVS, Figure 3, Figure S2), and was at a similar level compared to other European bat-associated lyssaviruses (Figure 3, insets). 

## 4. Discussion

After the detection of rabies in a bat from Finland, sequence analyses of the partial viral genome suggested that a novel lyssavirus was discovered [32]. For any novel lyssavirus with zoonotic potential, a risk assessment is needed that should elaborate whether vaccination using standard RABV vaccines would elicit an immune response that would also neutralize the new pathogen. For such characterizations, viable virus would be required since it was shown that antigenicity may not be derived simply from phylogeny [48]. Unfortunately, we were not able to isolate viable virus from the remaining bat carcass, neither in cell culture nor in-vivo. A high proportion of fragmented RNA indicated (Figure S1) that the bat was already in a decomposed state, thus hampering efforts to isolate virus. Nonetheless, using sophisticated enrichment strategies for high throughput sequencing as described before [36], we were able to generate the full genome of this novel virus, additionally to the identification of the potential host. Phylogenetically, KBLV clusters closely with BBLV, EBLV-2 and KHUV, suggesting the placement within phylogroup I. With a genetic identity below 80% in the N-gene and the full genome as compared to the closest known lyssaviruses, the demarcation criteria for placing KBLV as a novel lyssavirus species are met. It remains enigmatic, similarly to the recent discovery of BBLV [28], as to why KBLV had not been identified earlier, given that *M. brandtii* bats had been part of surveillance activities across Europe [22]. Previously known and closely related lyssaviruses from phylogroup I were also isolated from bat species of the genus Myotis (Figure 1), thus supporting that *M. brandtii* is likely a reservoir host and not infected as a result from a spill-over infection by another bat species, although this latter possibility cannot be excluded given the available data. The Myotis genus, which belongs to the family *Vespertilionidae* and comprises more than 100 species [49]. Interestingly, *M. brandtii* as a western Palaearctic bat species is actually part of the *Myotis* New World clade, suggesting that lyssavirus-bat host co-evolution occurred after the split between New and Old World within the *Myotis* radiation that occurred about 19 million years ago [50].

In the absence of viable virus, we used the viral genetic information combined with molecular methods to further characterize the virus. Initially, it was planned to clone and rescue a full length KBLV, but the different genes could not be cloned successfully into one plasmid. Also, in other attempts including the swapping of UTR and LTR with KHUV, the insertion of KBLV-G into recombinant BBLV and RABV, viable virus could not be rescued.

However, expression of the predicted KBLV-G from transfected plasmids (Figure 2) and successful complementation of G-gene deleted RABV by the KBLV G protein demonstrated functionality of the glycoprotein and allowed the production of KBLV-G pseudotyped RABV particles for neutralization assays. The latter demonstrated that sera from human vaccinees could neutralize KBLV (Figure 3, Figure S2), similarly to other phylogroup I lyssaviruses [47,51]. 

## 5. Conclusions

Functional assays based on molecular biology and sequence information were suitable to further characterize the novel lyssavirus KBLV in the absence of virus isolation. Further intensified surveillance targeting *M. brandtii* as the likely host are initiated to detect eventually another KBLV infected bat, thus increasing the chance of virus isolation. While our attempt to isolate replication competent KBLV or recombinant viruses did not yield viable virus, in the future, other avenues to rescue viruses may be more successful. 

All information on antigenic and genetic divergence fulfill the species demarcation criteria by ICTV [52], so that we propose KBLV as a separate species within the Lyssavirus genus. Phylogeny and cross-neutralization support that KBLV is another member of phylogroup I lyssaviruses, for which the classical RABV-based vaccines confer cross-protection as shown for EBLV-1, EBLV-2 and BBLV. Thus, KBLV represents only a minor public health risk that can be mitigated with recommendations on adequate behavior, e.g., to wear gloves when handling bats, preventive vaccination of bat-handlers and post exposure prophylactic treatment in humans exposed to bats.

## Data Availability

All data presented in this study are available in the supplementary material.

## Supplementary Materials

The following are available online at https://www.mdpi.com/1999-4915/13/1/69/s1, Table S1. Reference sequences of the 17 lyssavirus species included in the phylogenetic tree for the classification of the KBLV genome. Table S2. Genome organisation of the 17 known lyssavirus species and the new discovered Kotalahti bat lyssavirus. Table S3. RFFIT results of individual human sera using different test viruses for neutralization. Figure S1. Bioanalyzer 2100 RNA 6000 Pico chip measurement of the extracted RNA originating from the inoculum precipitate. Figure S2. Graph showing individual concentrations of virus neutralizing antibodies as measured using CVS and RABV pseudotyped with KBLV-G as test virus.
