# Supplementary material for: Genetic and Antigenetic Characterization of the Novel Kotalahti Bat Lyssavirus (KBLV)
Source: Viruses. 2021 Jan 6;13(1):69. doi: 10.3390/v13010069 (PMC7825429; doi:10.3390/v13010069)
Supplement: Supplementary file 1 [file viruses-13-00069-s001.pdf]

viruses

MDPI

Supplementary File

Genetic and antigenetic characterization of the novel Kotalahti bat lyssavirus (KBLV)

Table 1. Reference sequences of the 17 lyssavirus species included in the phylogenetic tree for the classification of the KBLV genome.

Lyssavirus species	Abbreviation	Accession number	Sequence length [b]
Aravan lyssavirus	ARAV	NC_020808.1	11918
Australian bat lyssavirus	ALBV	NC_003243.1	11822
Gannoruwa bat lyssavirus	GBLV	NC_031988.1	11919
Bokeloh bat lyssavirus	BBLV	NC_025251.1	11900
Khujand lyssavirus	KHUV	NC_025385.1	11903
European bat 1 lyssavirus	EBLV-1	NC_009527.1	11966
European bat 2 lyssavirus	EBLV-2	NC_009528.2	11930
Duvenhage lyssavirus	DUUV	NC_020810.1	11976
Taiwan bat lyssavirus	TWBLV	MF472710.1	11988
Irkut lyssavirus	IRKV	NC_020809.1	11980
Shimoni bat lyssavirus	SHIBV	NC_025365.1	12045
Mokola lyssavirus	MOKV	NC_006429.1	11940
Lledia bat lyssavirus	LLEBV	NC_031955.1	11931
West Caucasian bat lyssavirus	WCBV	NC_025377.1	12278
Lagos bat lyssavirus	LBV	NC_020807.1	12016
Ikoma lyssavirus	IKOV	NC_018629.1	11902
Rabies lyssavirus	RABV	NC_001542.1	11932

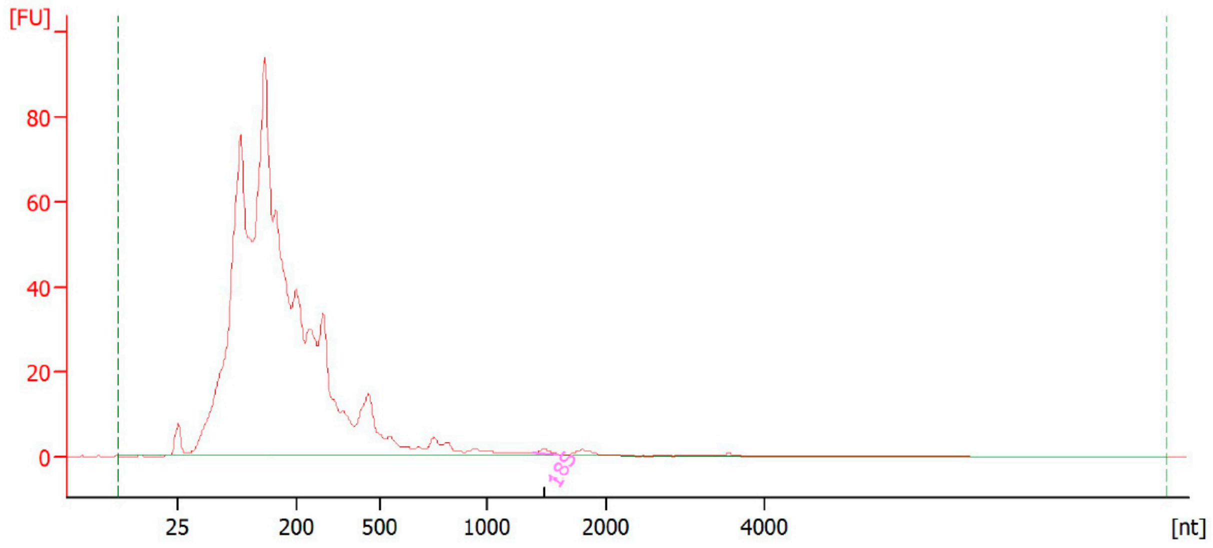

Figure 1. Bioanalyzer 2100 RNA 6000 Pico chip measurement of the extracted RNA originating from the inoculum precipitate. The high proportion of small RNA fragments indicates a high level of decomposition of the original sample material.

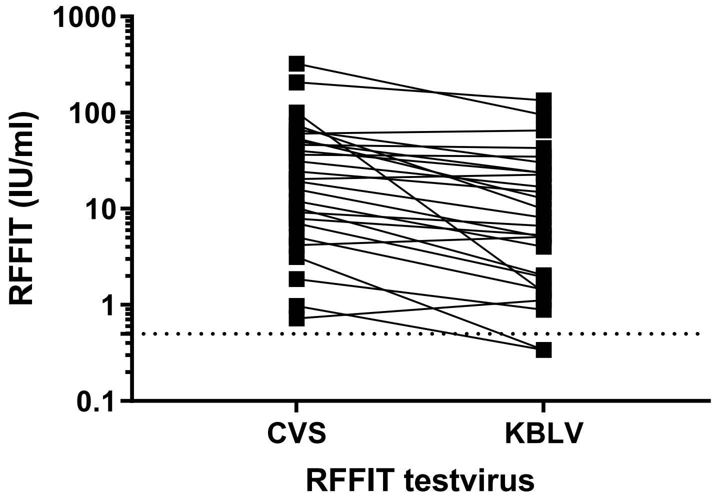

Figure 2. Graph showing individual concentrations of virus neutralizing antibodies as measured using CVS and RABV pseudotyped with KBLV-G as test virus. 0.5 IU/ml as the threshold for adequate immune response is indicated as dashed line.

Table 2. Genome organisation of the 17 known lyssavirus species and the new discovered Kotalahti bat lyssavirus.

	RABV	LBV	MOKV	DUVV	EBLV-1	EBLV-2	ABLV	ARAV	KHUV	IRKV	WCBV	SHIBV	BBLV	IKOV	GBLV	LLEBV	TWBLV	KBLV
3′UTR	70	70	70	70	70	70	70	70	70	07	70	70	70	70	70	70	70	70
**N protein**	1353	1353	1353	1356	1356	1356	1353	1356	1356	1356	1353	1353	1356	1353	1353	1353	1356	1356
N-P	90-91	101	100-102	90	90	101	94	85	95	93	64	98	91	66	92	68	99	86
**P protein**	894	918	912	897	897	894	894	894	894	897	894	918	894	870	894	870	897	894
P-M	88	75	80	83	83	88	89	85	72	82	133	76	86	74	88	74	82	63
**M protein**	609	609	609	609	609	609	609	609	609	609	609	609	609	609	609	609	609	609
M-G	211-5	204	203-204	191	211	210 (205)	207-209	210	208	214	206	205	210	209	212	198	212	210
**G protein**	1575	1569	1569	1602	1575	1575	1578-1581	1581	1581	1575	1578	1569	1575	1575	1581	1578	1659	1581
G-L	522	578-588	546-563	562-563	560	512	508-509	514	504	569	862	613	496	569	505	608	518	501
**L protein**	6384*	6384	6384	6384	6384	6384	6384	6384	6384	6384	6384	6384	6384	6381	6384	6381	6384	6384
5′UTR	131	145	112-114	130-131	131	131	131	130	130	131	125	150	129	126	131	122	70	124
**Genome**	11923-8	12006-16	11940-57	11975-6	11966	11930	11918	11918	11903	11980	12278	12045	11900	11902	11919	11931	11988	11878

Table 3. RFFIT results of human sera using different test viruses for neutralization. The titre was converted in international units (IU) per millilitre and log10 transformed.

Lab-ID	CVS	KBLV	EBLV-1	EBLV-2	BBLV
14448	2,32	2,13	2,13	2,26	1,96
14450	2,51	1,97	2,10	2,03	1,61
14459	1,87	1,00	1,26	1,27	0,12
14471	0,50	-0,47	-0,47	0,26	0,86
14476	0,27	-0,05	0,71	0,13	0,74
14477	0,96	0,82	0,28	0,51	0,52
14481	-0,01	-0,47	0,11	-0,06	0,42
14489	2,00	0,13	0,16	0,16	0,84
14491	1,56	1,54	1,08	1,05	1,12
14505	1,82	1,48	1,76	1,24	0,89
14742	1,67	1,63	1,28	1,23	1,58
14743	1,31	1,35	-0,88	0,89	1,09
14746	1,78	1,82	1,38	1,50	1,48
14755	0,90	0,72	0,72	0,80	0,37
14756	1,28	0,91	0,96	1,09	0,55
14759	-0,14	0,05	-0,34	-0,18	-0,75
15249	0,85	0,29	0,41	0,56	0,94
15255	1,08	0,60	0,80	0,62	0,88
15263	0,70	0,16	-0,02	0,43	0,44
15269	1,73	1,11	0,26	0,66	1,05
15270	1,01	0,31	0,30	0,68	0,82
15274	1,20	0,71	0,69	0,86	1,33
15275	1,39	1,17	1,14	1,10	0,92
15307	1,70	1,37	1,14	1,66	1,24
15315	1,61	1,38	1,33	1,49	1,43
15319	0,62	0,71	0,38	0,32	0,10
15325	1,49	1,22	0,96	1,09	1,00
